# Classic mechanisms and experimental models for the anti‐inflammatory effect of traditional Chinese medicine

**DOI:** 10.1002/ame2.12224

**Published:** 2022-04-12

**Authors:** Du Hongzhi, Hou Xiaoying, Guo Yujie, Chen Le, Miao Yuhuan, Liu Dahui, Huang Luqi

**Affiliations:** ^1^ National Resource Center for Chinese Materia Medica China Academy of Chinese Medical Sciences Beijing China; ^2^ Hubei Provincial Key Laboratory of Traditional Chinese Medicine Resources and Traditional Chinese Medicine Chemistry Hubei University of Chinese Medicine Wuhan China; ^3^ Wuhan Biomedical Research Institute, School of Medicine Jiang Han University Wuhan China

**Keywords:** anti‐inflammatory effect, inflammation and related diseases, the classical mechanisms, the experimental models, traditional Chinese medicine

## Abstract

Inflammation is a common disease involved in the pathogenesis, complications, and sequelae of a large number of related diseases, and therefore considerable research has been directed toward developing anti‐inflammatory drugs for the prevention and treatment of these diseases. Traditional Chinese medicine (TCM) has been used to treat inflammatory and related diseases since ancient times. According to the review of abundant modern scientific researches, it is suggested that TCM exhibit anti‐inflammatory effects at different levels, and via multiple pathways with various targets, and recently a series of in vitro and in vivo anti‐inflammatory models have been developed for anti‐inflammation research in TCM. Currently, the reported classic mechanisms of TCM and experimental models of its anti‐inflammatory effects provide reference points and guidance for further research and development of TCM. Importantly, the research clearly confirms that TCM is now and will continue to be an effective form of treatment for many types of inflammation and inflammation‐related diseases.

## Graphical abstract

Inflammatory is a common disease and involved in the pathogenesis, complications, as well as sequelae of a large number of related diseases. Therefore, people have been devoted to develop anti‐inflammatory drugs for the prevention and treatment of diseases. In fact, Traditional Chinese medicine (TCM) has been used to treat inflammatory and related diseases since ancient times. According to the review of abundant modern scientific researches, it is suggested that TCM exhibit anti‐inflammatory effect via different levels, multiple pathways and various targets. And a series of in vitro and in vivo anti‐inflammatory models have been developed for anti‐inflammatory research of TCM. Nowadays, these reported classical mechanisms and experimental models of anti‐inflammatory effect for TCM can provide reference and guidance for further research and development of TCM. Importantly, it is convinced that TCM is and will be an effective treatment for a variety of inflammation and inflammation‐related diseases.

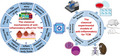

## 1 INTRODUCTION

Inflammation was one of the first diseases to be identified and diagnosed. More than 2000 years ago, the Roman physician Aulus Cornelius Celsus first described the classic symptoms of inflammation.^1^ Within the medical profession, inflammation is described as a complex defense response to injury in various cells, living tissues and the vascular system. Common inflammatory stimulants include physical factors (high heat, low temperature, UV, etc.), chemical factors (strong acids, strong bases, irritant solvents, etc.), mechanical factors (cutting, striking, squeezing, etc.), biological factors (parasites, bacteria, viruses, etc.) and immune factors (allergies and auto‐immune diseases).^2^ In other words, a huge number of factors in our lives may contribute to inflammation, with the result that inflammation is the most common disease or pathogenesis during our lifetime. An inflammatory response is initially a beneficial protective behavior of the human body. However, uncontrolled inflammation may lead to discomfort and damage to the body, and may even endanger life. For example, as is well known, when COVID‐19 is induced by SARS‐CoV‐2, the body produces an immediate inflammatory response. If not controlled in time, the coronavirus will ultimately cause overproduction of cytokines (known as a cytokine storm), damaging the tissues and organs seriously and even threating life.^2^, ^3^ Therefore, considerable research effort has been devoted to finding anti‐inflammatory drugs for the prevention and treatment of a variety of inflammatory diseases.

In 1880, aspirin may have been the first drug shown to be effective against inflammation, and subsequently hundreds of drugs have been approved to treat various inflammatory diseases. Generally, modern chemical and biological agents can be classically divided into non‐steroidal anti‐inflammatory drugs (NSAIDs) and steroidal anti‐inflammatory drugs (SAIDs). NSAIDs are one of the most common drugs used in daily life as treatments for colds, fever, headaches, etc. However, NSAIDs only relieve symptoms, and cannot eliminate the basic inflammatory factors and prevent the continued development of the disease.^4^ Thus, NSAIDs are generally used for mild inflammatory symptoms, while critical or severe diseases should be treated with SAIDs. SAIDs are mainly composed by glucocorticoids, which have a strong anti‐inflammatory effect. Unfortunately, SAIDs are a double‐edged sword, very easily causing adverse reactions that harm the human body and leave sequela.^5^ Because of the inevitable shortcomings of these traditional anti‐inflammatory drugs, researchers are exploring novel strategies with higher efficacies and lower toxicity to control inflammation. With the gradual modernization and international recognition of TCM, increasingly people acknowledge and accept Chinese medicine. Many TCM remedies, for example, Tripterygium wilfordii Hook.f.,^6^ Andrographis paniculata (Burm. f.) Nees,^7^ Coptis chinensis Franch,^8^ etc., have proved to have good anti‐inflammatory activity. Therefore, TCM is considered to be an effective anti‐inflammatory strategy.

In fact, TCM has been used to treat inflammatory diseases for thousands of years. Within the medical community it is widely believed that inflammation is involved in the pathogenesis, complications, and sequelae of many diseases,^9^, ^10^ and there is a great deal of evidence that the therapeutic effects of many TCMs are mediated by their anti‐inflammatory activities.^11^, ^12^ Thus, scientists around the world are developing drugs based on the anti‐inflammatory properties of TCMs. In 2015, Phynova Joint and Muscle Relief Tablets (*Siegesbeckiae* Herba extract) became the first Chinese medicine product to be approved by the MHRA (Medicines and Healthcare products Regulatory Agency) for marketing in the UK.^13^ And its anti‐inflammatory effect is the mechanism by which Phynova relieves joint and muscle pain. In summary, a crucial mechanism of TCM is its anti‐inflammatory action and TCM will prove to be an important effective treatment for inflammatory disease.

## 2 THE CLASSIC MECHANISMS OF THE ANTI‐INFLAMMATORY EFFECTS OF TRADITIONAL CHINESE MEDICINE

**FIGURE 1:**
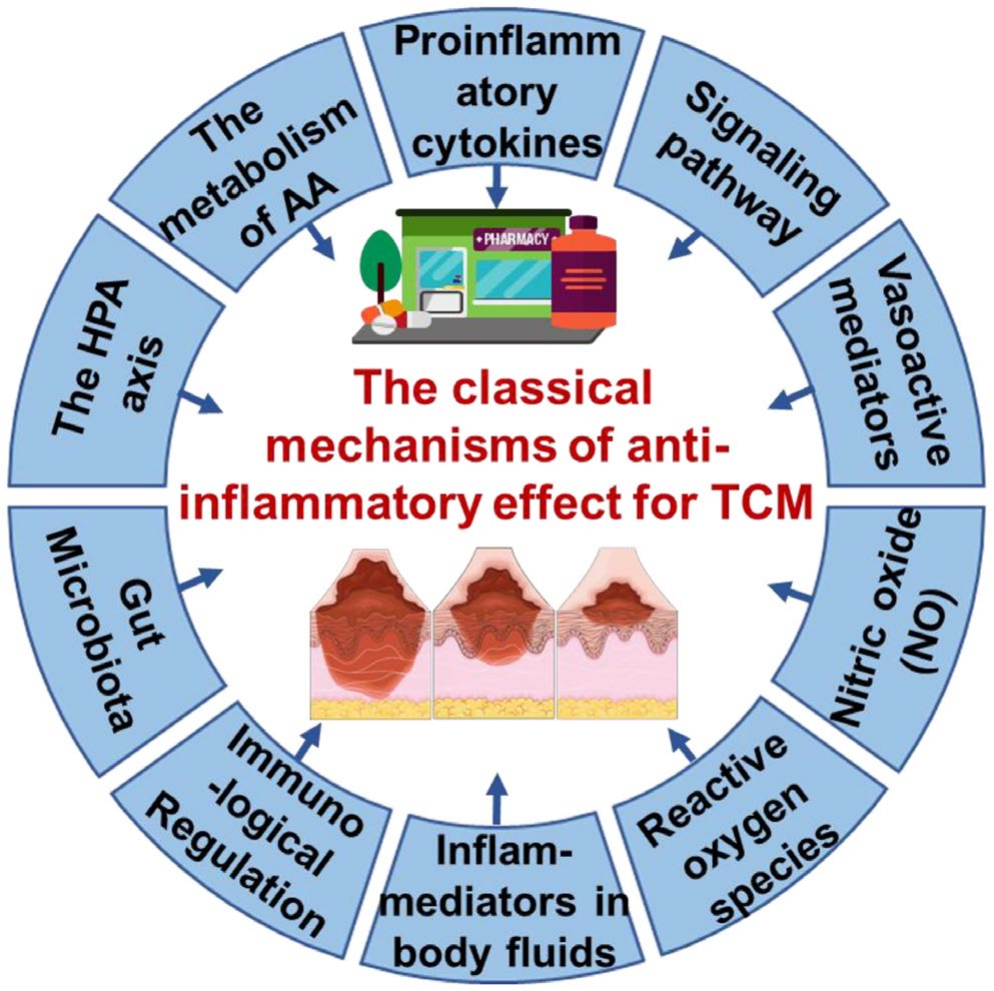
The classic mechanisms of the anti‐inflammatory effects of Traditional Chinese Medicine

The pathological mechanism of inflammation is a complex defense response involving diverse cells and various factors. The cells involved in the inflammatory response are phagocytes (mononuclear macrophages, neutrophils and eosinophils), platelets and endothelial cells, which are activated in response to inflammatory stimuli. After activation, the cells produce inflammatory mediators that initially protect the body by removing irritants but eventually develop into inflammatory diseases. Inflammation is usually divided into three distinct phases: it starts as increased vascular permeability, followed by infiltration of leukocytes, which eventually cause granuloma formation and tissue repair. Due to the complexity of its constituents, TCM exhibits anti‐inflammatory effects at different levels, via multiple pathways with various targets (Figure 1). Firstly, TCM may regulate the hypothalamic–pituitary–adrenal (HPA) axis via endogenous hormones to relieve inflammation. Then TCM could subsequently inhibit the production and release of inflammatory mediators and interfere with binding to receptors. At the same time, TCM could also counter oxidative stress and interact with multiple signaling pathways. In addition, TCM could activate the immune system to alleviate inflammation. In summary, the classic mechanisms of anti‐inflammatory effects of TCM are various and effective.

### 2.1 The hypothalamic–pituitary–adrenal (HPA) axis

A lot of TCMs exhibit glucocorticoid‐like pharmacological activity or regulate the function of HPA axis,^14^ ultimately leading to increased endogenous cortisol secretion, which exerts anti‐inflammatory effects. Thus, they are called HPA‐dependent anti‐inflammatory drugs. For instance, Gastrodiae Rhizoma water extract^15^ ameliorates inflammation via increased plasma corticosterone (CORT), adrenocorticotrophic hormone (ACTH), hypothalamic corticotropin‐releasing factor (CRF), and glucocorticoid receptor (GR) concentrations. CRF is a neuroregulatory factor found in the brain that regulates the transmission of serotonin (5‐HT), which is a key molecule in the occurrence and progression of inflammation and mainly regulates vascular permeability. Chinese herbal compound prescriptions could also exert anti‐inflammatory effects through the HPA axis. Bu‐Shen‐Yi‐Qi‐Tang relieves respiratory inflammation and inhibits hypothalamic–pituitary–adrenal axis activity in asthmatic mice.^16^ In addition, like SAIDs, TCM can exhibit anti‐inflammatory effects via increased endogenous cortisol secretion, but lacking the side effects of glucocorticoids, they can be more safely applied in clinical situations. Thus, its action on the HPA axis is an effective and significant mechanism by which TCM counteracts inflammation.

### 2.2 The metabolism of arachidonic acid (AA)

Arachidonic acid (AA) is an unsaturated arachidic acid catalyzed by phospholipase A2 (PLA2) after activation by inflammatory stimuli and inflammatory mediators.^17^ AA is then metabolized by cyclooxygenase (COX) and 5‐lipoxygenase (5‐LOX) pathways to produce various metabolites. Arachidonic acid metabolites – prostaglandins (PGs), leukotrienes (LTs), and 12‐hydroxyeicosatetraenoic acid (12‐HETE) – are actively involved in the development of various inflammatory diseases, like cancer, pneumonia and arthritis. Therefore, many of the key enzymes involved become important drug targets.^18^ Substantial evidence exists that TCM could target these enzymes to exert anti‐inflammatory effects.

#### 2.2.1 Phospholipase A2 (PLA2)

PLA2 is the rate‐limiting enzyme mediating the biosynthesis of AA from membrane phospholipids and is an extremely important target for drugs such as SAIDs. Several Chinese medicines also inhibit PLA2 to relieve inflammation. Scutellarin, a bioactive constituent in *Scutellaria baicalensis* Georgi, has been confirmed as a potent inhibitor of PLA2.^19^ Similarly, the world‐famous Chinese medicine Yunnan Baiyao exhibits anti‐inflammatory effects via regulation of the PLA2/AA metabolic pathway in an acute inflammation rat model.^20^ Thus, like steroidal anti‐inflammatory drugs, TCMs can exert anti‐inflammatory effects via PLA2, confirming the scientific explanation of their application in critical inflammatory diseases.

#### 2.2.2 Cyclooxygenase (COX)

Cyclooxygenase (COX; also known as prostaglandin endoperoxide synthase, PGHS) is composed of the isoenzymes COX‐1 and COX‐2. COX is a key rate‐limiting enzyme in PG synthesis, which can induce AA to produce various PGs and thromboxane A2 (TXA2), leading to various physiological and pathological effects. Thus, COX is an extremely important target for drugs such as NSAIDs. The classical NSAID aspirin is a potent inhibitor against COX. Aspirin was developed from willow bark as an anti‐inflammatory and analgesic drug.^21^ Similarly, TCM also exerts anti‐inflammatory effects through COX. As recently reported, a series of bioactive constituents in the Chinese medicine Huo‐Luo‐Xiao‐Ling Dan have been shown to be inhibitors of COX.^22^ Acetyl‐11‐keto‐β‐boswellic acid, β‐boswellic acid, acetyl‐α‐boswellic acid, acetyl‐β‐boswellic acid, and betulinic acid were COX‐1 selective inhibitors. Senkyunolide O and cryptotanshinone were COX‐2 selective inhibitors. Roburic acid and phenethyl‐trans‐ferulate inhibited COX‐1 and COX‐2 equally. In addition, a large number of TCMs have also been confirmed to exert anti‐inflammatory effects via COX.^23^, ^24^, ^25^ Thus, COX is clearly a significant target for Traditional Chinese medicines.

#### 2.2.3 5‐lipoxygenase (5‐LOX)

The enzyme 5‐lipoxygenase (5‐LOX) is a key enzyme catalyzing AA into leukotrienes (LTs). LTs are recognized mediators of inflammation and play important roles in many diseases, and therefore 5‐LOX is considered to be a classic target for anti‐inflammatory drugs. Resveratrol,^26^ found in many TCMs (*Polygonum cuspidatum* Sieb.et Zucc, *Ampelopsis japonica* [Thunb] Makino, *Morus alba* L. and so on) is a proven 5‐LOX inhibitor. Published reports have also shown that Yunnan Baiyao,^27^ Huanglian Jiedu Decoction^28^ and *Dendropanax dentiger* (Harms) Merr^29^ can inhibit 5‐LOX and exhibit anti‐inflammatory effects. Therefore, 5‐LOX is an obvious target for Traditional Chinese medicines.

#### 2.2.4 Prostaglandin (PG)

Via the catalyst prostaglandin H synthase (PGHS, also called COX), AA is successively transformed into the prostaglandin intermediate metabolites PGG2 and PGH2. Subsequently, downstream prostaglandin synthases – PGI2 synthases, PGE2 synthases, PGF2α synthases, PGD2 synthases and thromboxane A2 synthases – respectively catalyze the metabolites of various bioactive prostaglandins including PGI2, PGE2, PGF2α, PGD2, thromboxane A2 (TXA2). As is well known, the bioactive prostaglandins are common proinflammatory mediators and play a crucial role in a variety of inflammation and related diseases. Previous reports showed that levels of prostaglandins (PGI2, PGF2a, PGD2, PGE2, TAX2 and TXB2) were increased in a rheumatoid arthritis (RA) model, and Huo Luo Xiao Ling Dan could significantly reduce them to exert anti‐inflammatory effects.^30^ Similarly, a great deal of research has indicated that Shi‐Wei‐Ba‐Du‐Tang,^31^ *Mosla chinensis* Maxim. cv. Jiangxiangru^32^ and Pteryxin^33^ can inhibit PGE and reduce inflammation, and *Cyathula officinalis* Kuan and Timosaponin AIII (a steroidal saponin from *Anemarrhena asphodeloides* Bunge) can suppress TXA2 and exhibit anti‐inflammatory activities.^34^ Among prostaglandins, PGE2 and TXA2 are representative indexers for evaluating the anti‐inflammatory effects of TCM.

#### 2.2.5 Leukotrienes (LTs)

Via the catalyst 5‐LOX, AA is primarily transformed into 5‐hydroperoxyeicosatetraenoic acid (5‐HpETE). 5‐HpETE is extremely unstable and easily degrades into leukotriene A4 (LTA_4_). LTA_4_ is then catalyzed by leukotriene A4 hydrolase (LTA4H), and eventually modified into the stable isoforms LTB_4_ and LTC_4_. Subsequently, LTC_4_ can be transformed into LTD_4_, LTE_4_ and LTF_4_. Currently, LTB_4_, LTC_4_, LTD_4_, LTE_4_ and LTF_4_ are recognized inflammation mediators for various diseases. As the rate‐limiting enzyme in LT synthesis, LTA4H is the recognized target for drugs.^35^ A previous study^36^ discoveried a series of LTA4H inhibitors in TCM, including revandchinone 1 and revandchinone 4 in Rhei Radix et Rhizoma, tridecanoic acid, tetracosanoic acid and methyl eicosanoate in Notopterygii Rhizoma et Radix, montanic acid methyl ester and N‐docosanoyl‐O‐aminobenzoate in Genitana Macrophyllae Radix, and so on. To date, researchers have mainly evaluated the expression of leukotriene metabolites after treatment. It was showed that leukotriene metabolites (5‐HpETE, 5‐hydroxyeicosatetraenoic acid [5‐HETE], 8‐HETE, 12‐HETE, 15‐HETE, LTB_4_, LTC_4_ and LTE_4_) were enhanced in a rheumatoid arthritis (RA) model, and Huo Luo Xiao Ling Dan could significantly reduce them.^30^ Among these leukotriene metabolites, LTB_4_ may be one of the most powerful leukocyte chemokines causing tissue and organ damage, and it has been shown that LTB_4_ can also be suppressed by Chinese medicines such as Danggui‐Shaoyao‐San,^37^ Mahuang decoction,^38^ *Bidens bipinnata* L^39^ and so on. In summary, leukotrienes are also a key indicator in the evaluation of the anti‐inflammatory effects of TCM.

### 2.3 Proinflammatory cytokines

Cytokines are produced by activated lymphocytes and mononuclear macrophages; those produced by lymphocytes are named as lympho‐cytokines, and others produced by mononuclear macrophages are called mononuclear cytokines. Released by monocytes and macrophages, tumor necrosis factor‐α (TNF‐α), interleukin‐1 (IL‐1) and IL‐6 are the main cytokines mediating inflammation. They are classic proinflammatory cytokines and the most common markers of inflammation, thus they are detected in almost all inflammatory studies. As reported in numerous studies, TCM can exert anti‐inflammation effects via inhibition of the expression or/and release of these proinflammatory cytokines,^40^ by interfering with proinflammatory cytokine activated signaling pathways,^41^ enhancing the body's inherent immunity against proinflammatory cytokines,^42^ etc. Therefore, proinflammatory cytokines are a classic indicator for exploring the anti‐inflammatory effects of TCM.

#### 2.3.1 TNF‐α

TNF‐α is mainly released by mononuclear macrophages after stimulation by exogenous and endogenous factors. Exogenous factors include cell wall components (in particular, lipopolysaccharides, lipids A and muramyl dipeptide [MDP]) of gram‐negative bacteria, fungi and some viruses. Endogenous factors include interferon‐γ (IFN‐γ), IL‐1 and colony‐stimulating factor (CSF), and IL‐10 can also suppress TNF‐α. After release, TNF‐α binds to the TNF receptors (TNF‐R1 and TNF‐R2), activating multiple pathways including MAPK (JNK, ERK, P38), NF‐κb, PI3K‐AKT and apoptosis signaling pathways.^43^ Eventually, it causes platelet adhesion and aggregation, releasing histamine, increasing vascular permeability and affecting blood flow function. It also induces the production of proteolytic enzymes and oxygen radicals, damaging a wide range of cells and tissues. Therefore, anti‐inflammation drugs aim to inhibit the expression of TNF‐α or/and its binding with TNF receptor. There have been a large number of reports^40^, ^41^ that TNF‐α or/and TNF receptor are key components of the anti‐inflammatory effect of Chinese medicines (Qingfei Paidu decoction, Jinhua Qinggan Granule, Muscone and so on).

#### 2.3.2 IL‐1

IL‐1 is predominantly secreted by T lymphocytes, mononuclear macrophages and neutrophils. There are two different types of IL‐1 (IL‐1α and IL‐1β), which basically have the same effect as TNF‐α. IL‐1 binds to the IL‐1 receptor (IL‐1R), activating multiple pathways including the MAPK (MKK3/6, JNK, P38), NF‐κb, mTOR, MyD88, TRAF6, and TAK1(MAP3K7) signaling pathways.^44^ Likewise, IL‐1 and/or IL‐1R are also confirmed to play key roles in the anti‐inflammatory actions of many Chinese medicines (Huoxue Jiedu Huayu Formula and *Ophiopogon japonicus* [Linn. f.] Ker‐Gawl.).^45^, ^46^

#### 2.3.3 IL‐6

Released by monocytes, lymphocytes, endothelial cells and fibroblasts, IL‐6 can act on a variety of cells, and by linking to the IL‐6 receptor (IL‐6R), it activates multiple pathways including the JAK–STAT, PI3K‐Akt and MAPK signaling pathways.^47^ Moreover, IL‐6 also induces production of IL‐1 and TNF‐α. Eventually, these proinflammatory cytokines work together. As abundant reports show,^48^, ^49^ TCM (Xuanfei Baidu Decoction and Baitouweng decoction) can exert anti‐inflammatory effects by suppression of IL‐6.

#### 2.3.4 Other cytokines

In fact, there are dozens of proinflammatory cytokines in the body. In addition to the above‐mentioned cytokines, IL‐2, IL‐8, IL‐12, G‐CSF, GM‐CSF, MCP‐1, MMP‐1, MMP‐3 and IP‐10 have also proved to have inflammatory actions. For example, SARS‐CoV‐2 induced cytokine storms include not only IL‐1, IL‐6 and TNF‐α but also IL‐2, IL‐8, IL‐12, G‐CSF, GM‐CSF, MCP‐1 and IP‐10.^2^ It has been reported that TCM (Qingfei Paidu decoction, Huashi Baidu decoction, Lianhua Qingwen capsule, etc.) used in the treatment of COVID‐19 can suppress cytokine storms.^40^, ^50^

### 2.4 Signaling pathways

As shown above, AA metabolites and proinflammatory cytokines can activate multiple signaling pathways, further promoting the secretion of cytokines and affecting the normal function of cells and tissues. According to published research,^43^, ^44^, ^47^ MAPK, NF‐κb, PI3K‐AKT, JAK–STAT, mTOR, AMPK, apoptosis, MyD88 and so on are classical signaling pathways of inflammation. Further research may reveal more targets and pathways involved in inflammatory responses. As currently reported,^51^ one or more pathways may participate in the anti‐inflammatory effect of TCM. In fact, due to the complexity of Chinese medicine and the inter‐relationship of signaling pathways, TCM, especially Chinese medicine preparations, may affect most of the signaling pathways. Many outstanding studies using omics technology such as transcriptomics,^52^ proteomics^53^ and metabolomics have revealed the mechanisms of the anti‐inflammatory effects of TCM^54^ and show that it is possible that multiple pathways may be involved in these effects.

### 2.5 Vasoactive mediators

Vasoactive amines include histamine and 5‐hydroxytryptamine (5‐HT). Histamine produced by mast cells, eosinophils, and platelets after stimulation, can induce the contraction of endothelial cells and increase the permeability of vascular tissue, while 5‐HT, also called serotonin, is released by platelets after stimulation of the collagen and anti‐collagen antibody complex and platelet activating factor (PAF), which also increases the permeability of vascular tissue. In addition, PAF is released by basophils, neutrophils, monocytes, and endothelial cells. It can activate platelets, causing adhesion and aggregation of platelets and the release of vasoactive amines and also contributes to activating activate endothelial cells, increasing the permeability of vascular. PAF also stimulates the biosynthesis of other inflammatory mediators (especially PG and LT). Research has suggested that Chinese medicines such as Ginkgo diterpene lactones,^55^ Naoxintong Capsule^56^ and others can inhibit these vasoactive mediators.

### 2.6 Nitric oxide (NO)

NO is produced from L‐arginine by nitric oxide synthase (NOS). After the stimulation of LPS, TNF‐α, IL‐1 and IFN‐γ, mononuclear macrophages, polymorphonuclear leukocytes and vascular smooth muscle cells will enhance the secretion of NO to increase local blood flow and promote extravasation of plasma, which eventually forms edema. Moreover, NO also binds to key enzymes in the respiratory chain, causing enzyme inactivation, finally leading to cytotoxic effects and tissue damage. Therefore, NO is one of the classic indexes in evaluations of anti‐inflammatory activity of TCM in almost all studies.^32^, ^37^, ^57^

### 2.7 Reactive oxygen species (ROS)

Neutrophils and monocytes stimulated by inflammatory factors can release a series of reactive oxygen metabolites (superoxide anion free radicals, hydrogen peroxide, hydroxyl radicals and hypochlorous acid) via catalysis by multiple oxidases (NOX, SOD and MPO). As is will known, ROS can induce inflammation, inhibit the activity of various enzymes, and damage endothelial cells, resulting in increased permeability, and causing multicellular and tissue damage.^2^, ^58^ There is strong evidence that Chinese medicine can also exert an anti‐inflammatory effect through anti‐oxidation.

### 2.8 Inflammatory mediators in body fluids

In body fluids, there are three interrelated systems, namely complement, kinin and clotting, that are important inflammation mediators. In complement systems, C_3a_ and C_5a_ are key inflammation mediators that increase the permeability of vascular tissue, activate the metabolism of AA and promote the release of inflammatory mediators. In kinin systems, kinins increase the permeability of vascular tissue, contract smooth muscles, and evoke pain in the area of inflammation. In clotting systems, thrombin and fibrinolytic enzymes are related to the permeability of vascular tissue, leukocyte chemotaxis and vascular inflammation. Many studies^59^, ^60^ have shown that complement, kinin and clotting systems play a key role in the anti‐inflammatory effect of Chinese medicine.

### 2.9 Immunological regulation

Inflammation and immune responses are two dominating responses to foreign bodies, two sides of the same problem, but overlapping and inseparable. Recent progress in immunity research has shown the importance of immunity in controlling disease. Since ancient times, TCM has embraced a similar concept of immune regulation and it is accepted that immune regulation is an important mechanism for the pharmacological activity of Chinese medicine.^61^ As has been reported, Chinese medicines such as Moutan cortex radicis,^62^ Gegen Qinlian decoction,^42^ and others can ameliorate inflammatory diseases via immunological regulation. Therefore, immunological regulation is another classical mechanism of the anti‐inflammatory effect of TCM.

### 2.10 Gut microbiota

In recent years, the importance of gut microbiota in disease progression has increasingly been recognized. Gut microbiota are an indispensable part of the human body and their distribution and function are vital for human health. Disturbing the balance of gut microbiota leads to various diseases and is not conducive to controlling disease. Therefore, gut microbiota have become an important target for drug development. On the one hand, Chinese medicines are mostly taken orally into the digestive tract, and thus gut microbes can directly influence the efficacy of these medicines.^63^ In the gut, the ingredients in TCM can be metabolized and absorbed. For example, polysaccharides as the main components of many TCM^64^ (such as *Astragalus membranaceus* [Fisch.] Bunge., *Lycium chinense* Miller and *Panax ginseng* C. A. Meyer) should be decomposed into oligosaccharides and monosaccharides by gut microbiota and then absorbed into the blood circulation where they exhibit an anti‐inflammatory effect. On the other hand, many TCMs also affect the balance of gut microbiota. Dysbacteriosis has been proved to be the pathological mechanism of various inflammatory diseases, and TCM can reverse this situation. It has been confirmed that Danggui‐Shaoyao‐San improved hepatic lipid homeostasis via significant up‐regulation of the recognized probiotic Akkermansia,^65^ and Pai‐Nong‐San alleviated the development of colitis‐associated colorectal cancer by adjusting levels of *Firmicutes, Bacteroidetes, Proteobacteria*, and *Lactobacillus*.^66^ Intriguingly, modern regulation theory of gut microbiota (including a systematic perspective, balance, diverse microbiotas with the same or different functions) is similar to the traditional theory of TCM (a holistic view, harmony, various constituents with the same or different effects), so gut microbiota research is considered an excellent way to reveal the mechanisms of the anti‐inflammatory effects of TCM.^63^, ^67^

## 3 THE CLASSICAL EXPERIMENTAL MODEL OF THE ANTI‐INFLAMMATORY EFFECT OF TRADITIONAL CHINESE MEDICINE

**FIGURE 2:**
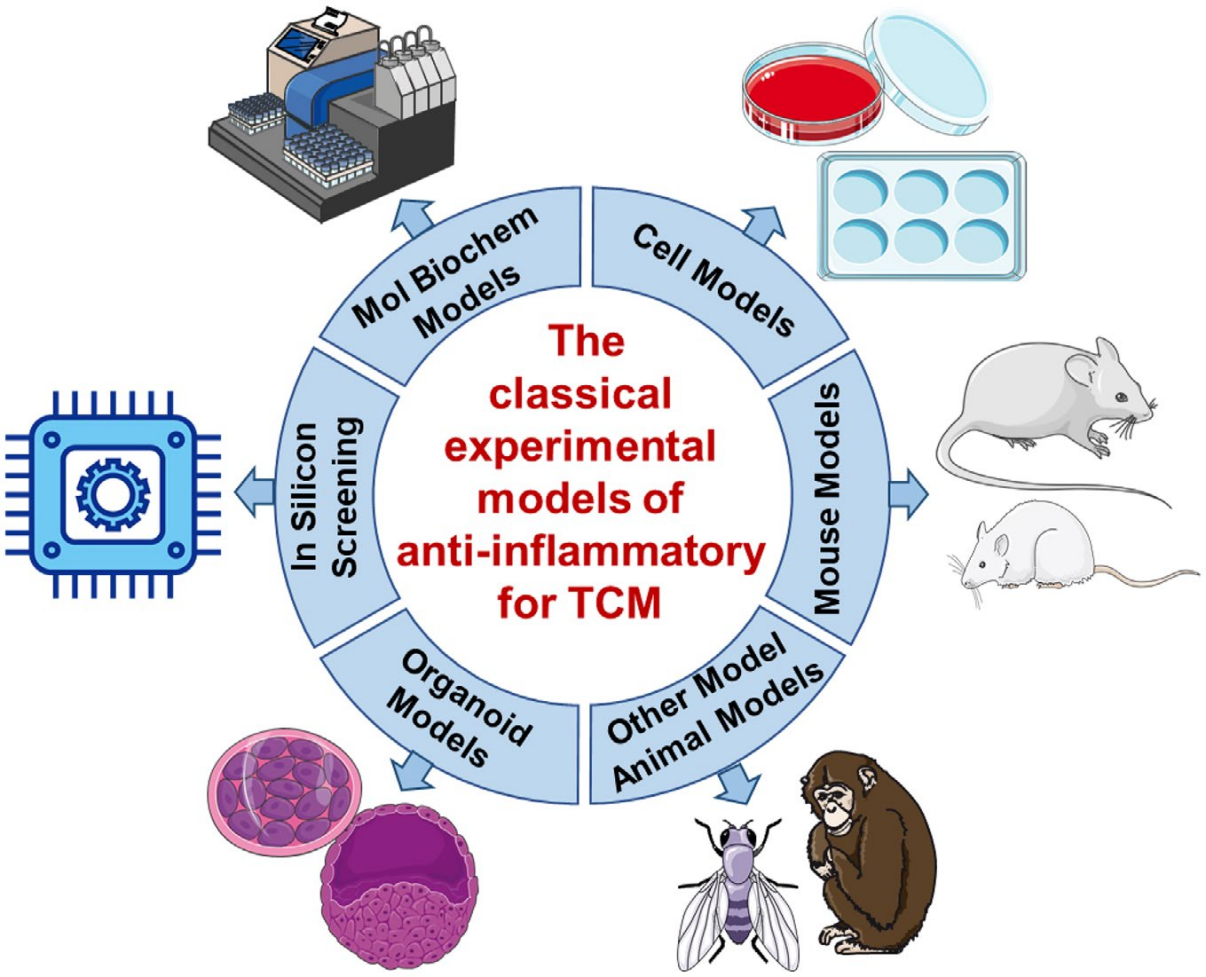
The classic experimental model of the anti‐inflammatory effects of Traditional Chinese Medicine

Inflammation is a common condition related to almost all diseases and thus much effort has been devoted to finding anti‐inflammatory drugs for the prevention and treatment of a variety of diseases. Due to the extensive clinical experience of TCM practitioners, Chinese medicine has become a source of new drug development. Although a large number of Chinese medicines with anti‐inflammatory effects have been used for thousands of years, it is essential to confirm the mechanisms of actions of TCM using modern pharmacological experiments. As stated above, anti‐inflammatory activity is an important aspect of TCM, and therefore a series of in vitro and in vivo anti‐inflammatory models are used to study TCM (Figure 2).

### 3.1 In silico screening

In silico screening consists of artificial intelligence drug screening, computer based virtual drug screening, molecular docking, network pharmacology and bioinformatics analysis. Currently, in silico screening is a commonly used model. Potential drugs targeting inflammation caused by COVID‐19 can be screened in a few dozen hours, and some TCM preparations have been assessed.^50^ In silico screening is thus an efficient model for evaluating anti‐inflammatory drug activity. In addition, in silico screening can quickly indicate potential mechanisms of the anti‐inflammatory effects of Chinese Medicine. Similarly, a large number of effective Chinese medicines were used to treat COVID‐19 in 2020, based on the basic theory and clinical experience of TCM, even though the scientific mechanism was unclear. Subsequently, researchers quickly determined the mechanism based on in silico screening, which provided scientific support for clinical application and a reference for subsequent research.^68^ Briefly, in silico screening is the fastest and cheapest model of assessing anti‐inflammatory drug activity, but the results need to be confirmed by other methods owing to its low level of accuracy.

### 3.2 Molecular and biochemical models

There are a large number of key enzymes involved in the occurrence and development of inflammation. A drug will have high potential as an anti‐inflammatory once it shows inhibitory activity on relevant enzymes. Thus, the construction of molecular and biochemical models for enzyme activity is of great importance. Classical molecular and biochemical models of PLA2, COX, 5‐LOX, PGE2 synthases, LTA4H and so on have been established for TCM.^19^, ^22^, ^32^, ^69^ Models for key targets of signaling pathways have also been established. Considering the close relationship between oxidative stress and inflammation, molecular and biochemical models of antioxidant activity (DPPH, ABTS and total antioxidant capacity assay) can also be used to evaluate anti‐inflammatory effects.^70^ At present, these molecular and biochemical models have been widely applied to test the anti‐inflammatory effects of TCM.^19^, ^22^, ^71^ Additionally, these models are also ideal for drug screening. In conclusion, molecular and biochemical models are more accurate than in silico screening, but the cell‐free system is also not very reliable when compared to cell‐based models.

### 3.3 Cell‐based models

Cell models are used to detect the changes in metabolites or products after exposure to TCM. Inflammatory factors (such as LPS, proinflammatory cytokines, etc.), stimulated cells (such as RAW 264.7, THP‐1, endothelial cell, etc.) and primary cells are common models to mimic inflammation. In cell models,^41^, ^72^, ^73^ researchers are able to evaluate the anti‐inflammatory effect of TCM by detection of the inflammatory mediators, such as proinflammatory cytokines, NO and key protein or enzymes, using methods such as enzyme‐linked immunosorbent assay (Elisa), reverse transcription‐polymerase chain reaction (RT‐PCR), western blot (WB), luciferase reporter gene assay (reporter system), and liquid chromatography–mass spectrometry technology (LC–MS). In addition, cell‐based models also can be applied in high throughput screening (cell‐based HTS),^74^ which may be more reliable than in silico screening and molecular and biochemical models. On the one hand, some active ingredients in TCM, such as scutellarin^19^ (*Scutellaria baicalensis* Georgi) and Eucalyptin C^75^ (*Eucalyptus globulus* Labill.), can directly affect the relevant targets to exert an anti‐inflammatory effect, which is reflected in the reversal of inflammation indicators. On the other hand, as reported in most current studies,^48^, ^72^ TCM may indirectly inhibit the expression of inflammation related mediators and targets, but the precise mechanism is not yet clear. In short, cell‐based models are the most classic methods currently among the methods for assessing the anti‐inflammatory effects of TCM.

### 3.4 Mouse models

Inflammation usually starts with increased vascular permeability, followed by infiltration of leukocytes, and eventually develops into granuloma and tissue repair. Therefore, the mediator‐induced inflammation mouse is one of the most reliable models for anti‐inflammatory drug research. Mouse models simulate the pathological features of inflammatory diseases better than cell models. Consistent with clinical practice, animal experiments are divided into acute and chronic inflammatory models. At present, both rat and mouse inflammatory models have been widely applied in the study of TCM.

#### 3.4.1 Acute inflammatory models

##### Chemical‐induced ear edema

Xylene, arachidonic acid, oxazolone, croton oil, 12‐O‐tetradecanoylphorbol‐13‐acetate (TPA) and phorbol myristate acetate (PMA) are commonly used as chemical inflammation inducers in mice.^73^, ^76^, ^77^ The inductive chemical can induce inflammation of the ear, which shows as ear edema. The anti‐inflammation effect can then be evaluated through measurement of the ear's thickness and weight, detection of inflammatory mediators (proinflammatory cytokines, NO and so on) and observation of tissue slices. Extract from *Ipomoea stolonifera* can inhibit ear edema and MPO activity induced by croton oil, showing remarkable anti‐Inflammatory activity.^73^ Similarly, the TPA induced ear edema model confirmed the anti‐inflammatory activity of *Sapium sebiferum* (L.) Roxb and suggested that ellagic acid, isoquercitrin and astragalin were the active ingredients by detection of SOD, CAT and GCL activities and the GSH content.^77^ Different chemical‐induced inflammatory markers may be discrepant, and one model may not tell the whole story. Considering the diversity of different Chinese medicines, it is better to adopt multiple models^73^ to evaluate the anti‐inflammation effect of Chinese medicine.

##### Chemical‐induced paw edema

Carrageenan, histamine, 5‐HT, bradykinin, dextran, and lipopolysaccharide (LPS) are commonly applied as chemical inducers in rats.^73^, ^76^, ^78^ After stimulation by these chemicals, scientists measure the paw withdrawal thermal latency (PWTL) using the hot plate test, the volume and thickness of edematous paw and the expression of inflammatory mediators, to explore the anti‐inflammation effect of TCM.^73^, ^78^ The anti‐inflammatory activity of Ipomoea stolonifera shown in the mouse ear edema model has been confirmed in the carrageenan‐induced rat paw edema model, which also showed the pharmaceutical effects.^73^ In the same model, the anti‐inflammation effect of Pudilan antiphlogistic oral liquid was revealed, providing scientific support for wide clinical application.^78^ Among the chemical‐induced paw edema models, the carrageenan induced paw edema model is the most widely used in research on the anti‐inflammatory effects of Chinese medicine.

##### Chemical‐induced vascular permeability

During inflammation, vascular permeability is elevated to permit antibodies and complement to access the infected or injured tissues. Acetic acid and Compound 48/80 are potent activators of histamine release, which increases vascular permeability through dilation of arterioles and venules. In this model, a dye (such as Evans blue) is injected into the tail vein of mice, and then acetic acid or Compound 48/80 is injected into the abdominal cavity. Eventually the anti‐inflammatory activity is evaluated by measuring the content of Evans blue in the abdominal fluid. TCMs such as, Lian‐Zhi‐San can significantly ameliorate Evans blue extravasation in an experimental hemorrhoidal model.^79^ The chemical‐induced vascular permeability model is thus another classic method for evaluating the anti‐inflammatory effect of Chinese medicine.

##### Chemical‐induced pleurisy models

Carrageenan, dextran and compound 48/80 are commonly used as chemical inducers.^76^ In these models, mice are pretreated with tested drugs or solvent 1 hour before the induction of pleural inflammation. Then carrageenan is injected into the pleura on the right side of the chest to induce pleurisy. Four hours later, the mice are sacrificed, dissected and analyzed. The volume of pleural exudate, the total number of white blood cells in the pleural exudate, the levels of inflammatory mediators (proinflammatory cytokines, NO and so on) and ROS related indexes in serum are measured to evaluate anti‐inflammatory activity. *Eriobotrya japonica* leaves presented anti‐inflammatory activity in a carrageenan‐induced pleurisy model, inhibiting leukocyte migration, protein extravasation and nitric oxide production.^80^ Chemical‐induced pleurisy models are also widely used in anti‐inflammatory research in Chinese medicine.

##### Systemic inflammation models

Severe inflammatory diseases, such as the inflammatory response caused by the SARS‐CoV‐2 virus, tend to be systemic, multi‐organ infections.^2^, ^50^ LPS, *Escherichia coli*, TNF‐α and zymosan are widely applied as chemical inducers for systemic inflammation models. After intraperitoneally or intravenously injection, the compounds activate a systemic inflammation response, promote inflammatory cytokine secretion, regulate inflammation related signaling pathways, release ROS and eventually cause multiple organ damage.^76^ Lianhua Qingwen (LHQW), the world‐famous TCM used against COVID‐19, could effectively treat sepsis‐induced acute lung injury in an LPS‐induced systemic inflammation model.^81^ Additionally, cecal ligation and puncture (CLP) also serves as a classical experimental model for systemic inflammation. CLP allows intestinal contents and bacteria to enter the abdominal cavity, causing abdominal infections and eventually systemic inflammation, and Shen‐Fu Decoction has been shown to ameliorate sepsis‐induced organic damage and mortality in a CLP model.^82^ Although systemic inflammation is intractable, there have been plentiful reports that TCM can effectively control systemic inflammation.

#### 3.4.2 Chronic inflammation models

##### Granuloma

As acute models do not adequately reflect the anti‐inflammatory effects of TCM, a chronic inflammation model may be more suitable for testing Chinese medicine. Granuloma can accurately represent the pathological progress of chronic inflammation.^73^, ^76^ Cotton pellets and glass rods are the classic inducers in rats.^73^ After sacrifice, the weight of granuloma, the expression of inflammatory mediators (proinflammatory cytokines, NO and so on) and the level of ROS related indexes are assessed to evaluate anti‐inflammatory activity. The anti‐inflammatory effect of *Taraxacum officinale*,^83^ *Ipomoea stolonifera* ^73^ and Qingdaisan (Formulated Indigo powder)^84^ has been confirmed in a cotton pellet‐induced granuloma model. Thus, granuloma is a widely used model for the assessment of chronic anti‐inflammatory activity of TCM.

##### Complete Freund's Adjuvant (CFA)‐induced arthritis

CFA contains heat‐inactivated mycobacterium tuberculosis, paraffin oil, dimannitol and oleic acid, which stimulates local inflammation.^73^, ^76^ After injection into the footpad of rats, CFA causes edema in periarticular tissues such as ligaments and joint capsules. Edema increases gradually during the early phase of the inflammation, rising to a constant level within 2 weeks. The anti‐inflammatory effect of TCM can be evaluated by measuring the volume and thickness of the edematous paw and the expression of inflammatory mediators, and radiological and histopathological analysis., The anti‐inflammatory effect of *Ipomoea stolonifera* has been consistently revealed in the chronic CFA‐induced arthritis model.^73^ Currently, the CFA‐induced arthritis model has been widely used to evaluate the anti‐inflammatory activity of TCM.

#### 3.4.3 Other inflammation‐related specific disease models

The acute and chronic inflammatory models described above have been widely employed in the early stages of anti‐inflammatory drug exploration, but inflammation‐related models of specific diseases should be built for further development and in‐depth research. For nervous system diseases, more appropriate models for inflammation‐related disease research include the intraventricular ameloid β1‐42‐injected AD rat model, the D‐galactose/sodium nitrite induced cognitive impairment model, and the global cerebral ischemia–reperfusion injury model, among others. For example, in the D‐galactose/NaNO_2_ induced cognitive impairment model, an effect of *Portulaca oleracea* in the prevention of aging and aging‐related cognitive dysfunction was suggested.^85^ For the digestive system, inflammation‐related models include the chemical induced gastritis model, the acetic acid induced recurrent aphthous ulcer, esoenteritis and others. *Citri Reticulatae Pericarpium* (Chenpi) is a traditional Chinese medicine for digestive system diseases, and its anti‐inflammation effect had been confirmed in a series of inflammation‐related specific disease models.^86^ Similarly, pneumonia, bronchitis and influenza are representative inflammation‐related models for the respiratory system. A significant curative effect of Lianhua Qingwen (LHQW) has been shown in multiple inflammation‐related specific disease models.^87^ In summary, there are also associated inflammatory models for potentially different diseases.

### 3.5 Other model animal models

Although rats and mice are the most widely used animal models of inflammation, monkeys, rabbits, zebrafish, drosophila and nematodes have also been used to study inflammation. Monkeys, the animal model best able to represent human pathology, have been employed to explore inflammation‐related diseases.^88^ Rabbits have also been used in anti‐inflammatory research on *Rhubarb*.^89^ As reported, tail amputation, LPS stimulation and copper sulfate exposure are the most common zebrafish models for inflammation research, and have been used in studies of schaftoside^90^ (the active constituent in *Artemisia Annua* L. *Artemisiae Argyi*, *Arisaema erubescens* [Wall.] Schott. and others) and indolealkylamines^91^ (from *Veneum Bufonis*). Drosophila has also been used in anti‐inflammation research on Chinese medicine.^90^ In addition, a few studies of the effects of Chinese medicine (*Polygonum multiflorum Thun*b extract) on inflammation also used *Caenorhabditis elegans* models.^91^ Experimental models of the anti‐inflammatory effects of TCM are clearly not limited to rodents, and other model animal models also contribute to inflammatory disease assessment.

### 3.6 Organoid models

With the continuing progress of science and technology, more and more new technologies have been applied in the study of inflammation. In particular, organoid models are considered to be the closest in vitro model to simulate the in vivo physiological and pathological characteristics of diseases.^92^ At present, organoid models of the intestines, lung, liver and stomach have been widely applied in the study of inflammation. As previously reported, organoid models have been used to evaluate the protective effect of TCM on intestinal damage (Glycyrrhetinic acid from *Glycyrrhiza uralensis* Fisch.),^93^ cancer (Gambogic acid from *Garcinia hamburgy* Hook. f.),^94^ regeneration of intestinal epithelia (*Trillium tschonoskii*)^95^ and so on. While research directly utilizing organoids for testing anti‐inflammatory effects of TCM is still lacking, we firmly believe that organoids will also become a recognized experimental model for future research on TCM.

## 4 CONCLUSION

Inflammation is a common condition related to almost all diseases. TCM has been used to treat inflammatory diseases for thousands of years. A great deal of evidence as proved that its anti‐inflammatory effect may be one of the important mechanisms of TCM.^71^ The pathological mechanisms of inflammation are complex, involving a variety of cells and factors. TCM can inhibit inflammation at different levels, via multiple pathways with various targets. To date, TCM has been shown to exert an anti‐inflammatory effect via well‐known mechanisms including the HPA axis, the metabolism of AA, proinflammatory cytokines, signaling pathways, vasoactive mediators, NO, ROS, inflammatory mediators in body fluids, immunological regulation and so on. In other words, the complexity of inflammation, involving multiple targets and pathways, and the holistic treatment theory of TCM, involving multiple components and mechanisms, mean that a specific TCM can exert its effect through multiple mechanisms, and even have an effect on all of these reported mechanisms.

Considering the complexity of the anti‐inflammatory mechanisms of TCM, further research requires the use of mature modern medical research strategies. In particular, the application of omics (Transcriptome, Proteome, Metabolome and Microbiome) technology could be used to objectively reveal the overall mechanisms of TCM's anti‐inflammation effects, rather than focusing on certain mechanisms. Omics techniques can be used alone or in combination to provide preliminary insights into mechanistic research, while ultimately the mechanisms identified should to be validated by conventional biochemical and molecular assessment. In addition, new technologies such as target fishing technology, network pharmacology and artificial intelligence are also helping us to explore the mechanisms behind the anti‐inflammatory effects of TCM. In short, a variety of reliable techniques should be broadly applied to objectively reveal the mechanisms of TCM's anti‐inflammatory effects.

For more specific experimental research, the classic experimental models including in silico screening, molecular and biochemical models, cell models, mouse models (acute and chronic inflammatory models) and other animal models can be used to evaluate the anti‐inflammatory effects of TCM, while organoid models provide a novel potential model. It can be seen that there are abundant models for anti‐inflammation research on TCM, and it is important to choose the appropriate model for the research project. First, use of a single model only is not reliable, and multiple model studies are more persuasive. Second, while in silico screening, molecular and biochemical models, cell models are suitable for drug screening or preliminary evaluation, the final evaluation and mechanistic study should be conducted in vivo. And last, the right animal model must be chosen to accord with the drug indication for specific Chinese medicines. The optimal research strategy is to verify the mechanism in clinical studies. In conclusion, TCM is and will continue to be an effective treatment for a variety of inflammation and inflammation‐related diseases.

## AUTHOR CONTRIBUTIONS

H.L. and L.D. designed and supervised the manuscript. D.H. and H.X. wrote the manuscript. G.Y., C.L. and M.Y. revised the manuscript. All authors read and approved the final manuscript.
